# Fertility preservation in women with endometriosis: Number 10 - October 2021

**DOI:** 10.1055/s-0041-1739234

**Published:** 2021-11-16

**Authors:** Márcia Mendonça Carneiro, João Sabino Lahorgue da Cunha Filho, Carlos Alberto Petta, Carlos Augusto Pires Costa Lino, Corival Lisboa Alves de Castro, Eduardo Schor, João Nogueira Neto, Marco Aurélio Pinho de Oliveira, Marcos Tcherniakovsky, Maurício Simões Abrão, Omero Benedicto Poli Neto, Ricardo de Almeida Quintairos, Sidney Pearce, Helizabet Salomão Abdalla, Julio Cesar Rosa e Silva

**Affiliations:** 1 Departamento de Ginecologia e Obstetrícia, Faculdade de Medicina, Universidade Federal de Minas Gerais, Belo Horizonte, MG, Brazil; 2 Departamento de Ginecologia e Obstetrícia, Universidade Federal do Rio Grande do Sul, Porto Alegre, RS, Brazil; 3 Universidade Estadual de Campinas, Campinas, SP, Brazil. Clínica Fertilidade & Vida, Campinas, SP, Brazil. Serviço de Reprodução Assistida, Hospital Sírio-Libanês, São Paulo, SP, Brazil; 4 Hospital Aliança, Salvador, BA, Brazil. Instituto de Perinatologia da Bahia, Salvador, BA, Brazil; 5 Hospital Geral de Goiânia, Goiânia, GO, Brazil; 6 Escola Paulista de Medicina, Universidade Federal de São Paulo, São Paulo, SP, Brazil. Sociedade Brazileira de Endometriose e Cirurgia Minimamente Invasiva; 7 Universidade Federal do Maranhão, São Luís, MA, Brazil; 8 Faculdade de Ciências Médicas, Universidade do Estado do Rio de Janeiro, Rio de Janeiro, RJ, Brazil; 9 Setor de Videoendoscopia Ginecológica e Endometriose, Faculdade de Medicina do ABC, Santo André, SP, Brazil; 10 Divisão de Ginecologia, Hospital Beneficência Portuguesa de São Paulo, São Paulo, SP, Brazil. Departamento de Ginecologia e Obstetrícia, Faculdade de Medicina, Universidade de São Paulo, São Paulo, SP, Brazil; 11 Departamento de Ginecologia e Obstetrícia, Faculdade de Medicina de Ribeirão Preto, Universidade de São Paulo, Ribeirão Preto, SP, Brazil; 12 Núcleo de Endometriose, Hospital Porto Dias, Belém, PA, Brazil; 13 Centro Universitário Christus, Fortaleza, CE, Brazil; 14 Faculdade de Ciências Médicas, Santa Casa de São Paulo, São Paulo, SP, Brazil; 15 Departamento de Ginecologia e Obstetrícia, Faculdade de Medicina de Ribeirão Preto, Universidade de São Paulo, Ribeirão Preto, SP, Brazil

## Key points

- Endometriosis is a common benign disease that can compromise female fertility.
- Fertility preservation is a key consideration in the care of girls and women with endometriosis, especially those with ovarian endometriomas.
- Although there is no definitive study on the subject yet, correct information on disease progression, treatment options, and the risks involved should be available for these women.
- It is too early to define fertility preservation as the standard of care for all women with endometriosis.
- Fertility preservation, however, should be considered for women with unoperated bilateral endometriomas and those who have previously removed unilateral endometriomas and need surgery for a contralateral recurrence.
- Since age is the most important prognostic factor, all patients should be aware of its adverse effect on fertility and pregnancy.
- Available strategies include cryopreservation of embryos and oocytes.
- Women should be counseled individually about the risks, benefits and costs involved. In this scenario, the approach of a multidisciplinary team on endometriosis is essential to reach good results.

## Recommendations

- Endometriosis is a benign disease that affects women during menacme and can adversely affect their fertility. The relationship between endometriosis and infertility is quite complex and remains unclear, therefore correct information about disease progression, treatment options and the risks involved should be available for these women.
- Several mechanisms are responsible for infertility in women with endometriosis, including a high production of cytokines and chemokines, an altered hormonal environment, increased oxidative stress, and compromised tubal and sperm function. Furthermore, endometriomas can interfere with folliculogenesis.
- It is too early to define fertility preservation as the standard of care for all women with endometriosis. However, this should be an important point to be considered in the care of girls and women with endometriosis, especially those with ovarian endometriomas.
- Women with unoperated bilateral endometriomas and those who have previously had unilateral endometriomas removed and need surgery for a contralateral recurrence should be advised on fertility preservation and available strategies, which include embryo and oocyte cryopreservation.
- Since age is the most important prognostic factor, all patients should be aware of its effect on their fertility plans.
- Women should be counseled individually about the risks, benefits and costs involved. In this scenario, the approach by a multidisciplinary team of endometriosis is a fundamental step for successful results.

## Clinical context

Fertility preservation is a topic that has attracted more attention from physicians and patients in recent years. 1 The increase in life expectancy and the possibility of cure achieved with advances in cancer treatments worldwide have made reproductive care and motherhood an important issue for young women undergoing cancer treatment. Likewise, women with other benign medical conditions, or for social reasons (postponement of motherhood), have now turned their attention to fertility. 2 3 4 The development of techniques that allow cryopreservation of oocytes has opened new perspectives to maintain these women's reproductive potential. 1

The topic is relevant for women with endometriosis, a condition that affects about 10% of women of reproductive age and up to 50% of women with chronic pelvic pain and infertility and who may have their ovarian reserve and future fertility compromised by the disease. 3 4 Several pathophysiological mechanisms can explain the occurrence of infertility in women with endometriosis, namely adnexal adhesions, tubal obstruction, hormonal imbalances, oocyte dysfunction, endometrial changes, inflammations that interfere with sperm-oocyte interaction, worse embryonic quality, lower implantation rate and decreased ovarian reserve. 3 Studies have shown that endometriosis may be associated with decreased ovarian reserve and a smaller number of oocytes in assisted reproduction technique (ART) treatments. 5 Apparently, in some cases, an interaction between the numerous pathophysiological changes may act through mechanisms not fully elucidated yet. 3

The exact effect of endometriosis on ovarian reserve remains to be established. The presence of ovarian endometriomas appears to adversely affect ovarian reserve markers, such as the anti-Mullerian hormone (AMH) by affecting its production or producing a direct effect not yet known. 6 In addition, large endometriomas can interfere with ovarian vascularization, and the treatment of endometriosis often requires surgery, particularly in patients with ovarian cysts and deep endometriosis. Repeated ovarian surgeries can lead to reduced ovarian reserve and even premature ovarian failure, as the healthy ovarian tissue, in part, ends up being excised along with the disease capsule. 7

Therefore, fertility preservation has become a relevant issue for women with endometriosis, especially those undergoing surgery for ovarian cysts. In this setting, these women should be appropriately advised about fertility issues prior to the procedure and receive evidence-based information about disease progression, ovarian reserve, available therapeutic options and the risks involved. 7 8

Cryopreservation of oocytes and embryos is an established fertility preservation technique that requires controlled ovarian hyperstimulation (COH) and follicle puncture for oocyte retrieval. Other techniques such as collection of immature oocytes followed by in vitro maturation for cryopreservation and cryopreservation of ovarian tissue were also studied. 2 8

Cryopreservation of ovarian tissue during surgery for endometriosis, previously considered an experimental practice, is already being routinely used in several countries. Thus, it is no longer experimental, becoming an interesting option for patients with endometriosis and surgical indication. 1

## Topic discussion

Fertility preservation is a relevant issue for women with endometriosis, and gynecologists should take it into account whenever evaluating these women. Key points to consider include the assessment of ovarian reserve, possible effects of surgery on fertility and the available fertility preservation options. 4 8 Oocyte cryopreservation should be routinely offered to women with endometriosis and infertility, who are at greater risk of needing in vitro fertilization (IVF) in the future. In this context, important issues to be discussed include how to approach young women with endometriosis, the possible effects of surgery on fertility, and the available options for fertility preservation. 9

## How to assess ovarian reserve?

The progressive loss of ovarian follicles is often responsible for subfertility and may also impact negatively on results obtained with the use of ARTs. Such loss is important for women currently not trying to become pregnant, but interested in preserving the chances of future pregnancy. 7 The assessment of ovarian reserve is an essential step in the treatment of women with endometriosis, especially those who will undergo infertility treatment thus, it should support and guide physicians with regard to fertility preservation. The presence of endometriotic lesions and cysts and the surgical procedures to treat these women may put the ovarian reserve at risk and reduce the number of oocytes available for the performance of ARTs. 3 5 6

The woman's age is the most important predictor of success with ARTs, given the reduction in pregnancy rates with advancing age. 9 10 Therefore, ovarian reserve markers should be evaluated to better inform patients about the expected success rates before starting any fertility-preserving procedure or surgery to treat endometriosis. Available tests include hormonal dosages of follicle-stimulating hormone (FSH) in the follicular phase and AMH, in addition to antral follicle count (AFC) and ovarian volume estimated by transvaginal ultrasound. Such tests can predict the number of oocytes obtained after COH and are related to pregnancy rates. 9 10

The ideal marker would be able to reflect a significant change throughout a woman's reproductive life, with significant shift from adolescence levels to the late reproductive period, allow the age-independent prediction of a woman's reproductive life span, in addition to spontaneous pregnancy in the general population. 9 10

Antral follicle count and AMH are the most reliable and used ovarian reserve markers. The AFC consists of counting the number of follicles with a diameter ranging from 2 to 10 mm and is widely used in ART clinics due to its ready availability and ease of evaluation. It correlates well with the response to ovarian hyperstimulation with gonadotropins. 9 10

The presence of ovarian endometriosis is associated with lower serum AMH, lower AFC, lower response to COH, and higher doses of gonadotropins used in ART cycles. Reduced ovarian reserve has been reported not only in women with ovarian endometriomas, but also in those with minimal to mild disease. 9

As excised endometriomas have oocytes firmly attached to the cyst wall, damage to the ovarian reserve is an important concern in endometriotic cyst surgery. There is a 2.4% risk of ovarian failure after bilateral ovarian endometrioma excision. Cystectomy can also have negative effects on ovarian blood supply and spontaneous ovulation rates. The impact of cystectomy on ovarian reserve can be reliably assessed by serum AMH measurements. 11

Despite the assumption that cyst drainage and wall ablation may be less harmful to ovarian reserve, they are associated with a lower chance of symptom reduction, lower pregnancy rates and higher endometrioma recurrence rates, so they are not recommended as first choice procedure. 11 12

Thus, patients considering pregnancy should not undergo repetitive ovarian preservation surgeries and minimize damage to the follicular reserve. 12 For those who do not plan to become pregnant immediately, a fertility-preserving approach should be considered before endometrioma surgery, or even during surgeries for advanced endometriosis. Martyn et al. 9 believe that AMH dosage should be offered to all women in their 30s who are not thinking about becoming pregnant, as the clinical evaluation will identify only about 50% of women at risk of reduced ovarian reserve. 2 13

## How should counseling about effects of surgery on future fertility be?

Pain relief and fertility improvement are the main goals of surgical treatment in women with endometriosis. Removing the disease while maintaining reproductive potential with minimal damage to reproductive organs remains a challenge in superficial, ovarian or deep endometriosis. 12

## What is the role of ovarian endometrioma surgery?

In the therapeutic planning of women who wish to maintain their reproductive potential, it is extremely important to take into account that the presence of endometriosis in any of its forms - superficial, ovarian or deep - can interfere with ovarian function and the endometrioma surgery may aggravate this situation. 3 5 7

Superficial endometriosis is associated with lower fertility rates and reduced ovarian reserve with low levels of AMH. 5 The presence of endometrioma also affects ovarian function, although the relationship between endometriomas and ovarian reserve damage remains controversial. The spontaneous ovulation rate is lower in the ovary with endometrioma. Follicular density is lower and fibrosis is more frequent in the ovarian cortex containing endometriomas. 14 In addition, the presence of deep endometriosis may be associated with reduced ovarian reserve and fewer oocytes retrieved in IVF cycles, probably due to the pelvic inflammatory process found in deep endometriosis. 14

Endometrioma surgery reduces follicular reserve with compromised ovarian function. This was demonstrated by the significant decrease in serum AMH levels after cystectomy and the decrease in ovulation rates after laparoscopic cystectomy compared to rates before surgery. 13 The decrease in AMH is greater in bilateral cystectomy compared to unilateral cystectomy. In IVF cycles, a lower number of oocytes was obtained with a decrease in the rates of pregnancies and live births after bilateral cystectomy, compared to cycles without endometriomas. 11 12 Muzii et al. 6 published a meta-analysis in which AFC was used to assess the effect of endometrioma surgery on ovarian reserve and reported that it did not decrease after endometrioma removal. However, as repeated surgeries to remove endometriomas seem to be more damaging to the ovarian reserve, indications for surgical treatment of endometrioma recurrence should be carefully evaluated. 11

Obviously, the larger the ovarian endometriomas and the more extensive and complex the pelvic adhesions, the worse the reproductive prognosis, and it is the surgeon's responsibility not to aggravate this situation. The fundamental principles governing these objectives are the preservation of ovarian follicular reserve and prevention of postoperative pelvic adhesions with a minimum possibility of residual disease. 11 12

## What is the role of deep endometriosis surgery in infertility?

Deep endometriosis is a specific entity arbitrarily defined in histological terms as endometriotic lesions extending more than 5 mm below the peritoneum, usually responsible for painful symptoms. Although deep endometriosis is often associated with infertility, the evidence for a clear connection between the disease and infertility is weak. Studies suggest that infertility in these women is likely due to the strong link between deep endometriosis and adhesions, superficial endometriotic implants, ovarian endometriomas, and adenomyosis. 15

Despite the evidence of the association between deep endometriosis and infertility, it is still unclear if surgery to treat this form of the disease can act on fertility, since the main indication of surgical approach was for the treatment of pelvic pain. 12 15

While some advocate complete surgical removal of endometriotic lesions for fertility improvement, others argue that extensive deep endometriosis surgery and intraperitoneal surgery in infertile women do not improve overall fertility prognosis and may be associated with a higher rate of complications. 15 16

In summary, the effect of surgery on the fertility of women with deep endometriosis remains unanswered given the heterogeneous nature of the disease and the lack of reliable trials with sufficient power and follow-up to study the topic. 16

## What are the fertility preservation options available?

Embryo cryopreservation and mature oocyte cryopreservation are established techniques to preserve fertility in women during the reproductive period. 17 In both cases, COH is required, followed by oocyte recovery with transvaginal ultrasound. The mature oocytes obtained can be cryopreserved or fertilized, and the resulting embryos cryopreserved. Embryo cryopreservation is an effective option, provided there is time to perform ovarian stimulation and an available partner. Oocyte cryopreservation is the best option for preserving fertility in women with endometriosis who wish to postpone pregnancy or will undergo surgical treatment for endometriosis and do not have a partner yet. 17 Vitrification appears to be an efficient method for cryopreservation of oocytes, keeping fertilization and pregnancy rates similar to those of IVF techniques with fresh oocytes. 18

There is concern about the quality of response in cases of endometriosis, as some studies suggest that women with endometriosis who undergo IVF cycles have lower rates of pregnancy and implantation compared to those with tubal infertility. 3 This would occur as a result of the reduced quality of oocytes, embryonic development and endometrial receptivity. Harb et al. 19 published a meta-analysis showing reduced rates of fertilization in women with grade I/II endometriosis, and of pregnancy and implantation in women with grade III/IV endometriosis. Therefore, more cycles of COH and IVF may be needed to obtain enough good quality oocytes to generate embryos with appropriate development and quality for freezing. Ovarian hyperstimulation does not seem to increase the risk of progression of endometriosis or recurrence of lesions in treated patients. 20 In addition, the presence of endometrioma at the time of ovum collection may increase the risk of pelvic infection and abscess formation. 11 12 21

Surgical approach should be carefully discussed in infertile patients with ovarian endometrioma. Excision of the endometrioma capsule increases the rate of spontaneous pregnancy in the postoperative period compared to drainage and electrocoagulation of the endometrioma wall. 22 However, these surgical techniques may present a risk of decreased ovarian reserve, either by removal of normal ovarian tissue during excision or by thermal damage to the ovarian cortex during ablation. Published data show a significant reduction in AMH values in the presence of endometriomas compared to the absence of endometriosis. 7 Surgical excision of endometriomas appears to negatively influence ovarian reserve, but only temporarily. 22 23 Other data suggest that the mere presence of an endometrioma adversely affects the ovarian reserve and it may be difficult to measure these effects before surgery. 22 23 Therefore, despite the efforts of laparoscopic surgeons to minimize surgical damage, the ovarian reserve can still suffer in the presence of endometrioma by itself. The size of the endometrioma, the risk of bilaterality of subsequent ovarian failure, the surgical technique, the surgeon's experience, and the patient's age should also be taken into consideration before surgical excision if future fertility is a concern. 22 23

Currently, cryopreservation of ovarian tissue is used for fertility preservation in women in reproductive years at high risk of losing ovarian function (chemotherapy, radiotherapy, or benign conditions associated with a high risk of premature ovarian failure). 1 17 In prepubescent girls at risk of losing their reproductive potential, this may be the only available alternative. However, note that cryopreservation of ovarian tissue is still considered experimental (Ethics Committee of the American Society for Reproductive Medicine, 2014; Decanter et al., 2018). 1 24 In patients with endometriosis, healthy fragments of the ovarian cortex can be isolated and cryopreserved during surgical removal of endometrioma. The technique should be carefully evaluated, given the risk of transferring small foci of endometriosis in the cryopreserved tissue. 17 24 The advantage of tissue cryopreservation is that there is no need for ovarian hyperstimulation. Many unanswered technical questions remain related to the choice of cryopreservation technique, chances of recovery of ovarian function after transplantation, and pregnancy rates after the procedure. 17 24 Data are still scarce regarding the use of this fertility preservation technique in women with endometriosis and further studies need to be conducted before indicating cryopreservation of ovarian tissue as the first choice for fertility preservation in patients with endometriosis. 24

**Figure 1.**
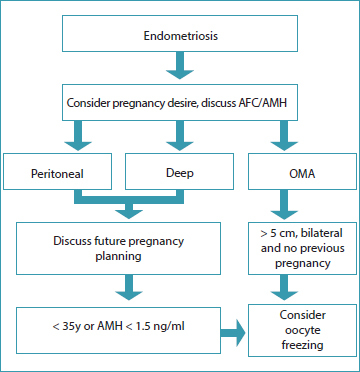
Evaluation flowchart for fertility preservation in women with endometriosis

Figure 1 summarizes the suggested assessment for fertility preservation in women with endometriosis.

## Final considerations

Endometriosis is a common benign disease that carries significant risks to reproductive organs. Fertility preservation is a key consideration in the care of girls and women with endometriosis, especially those with ovarian endometriomas and advanced age. Although there is no published prospective cohort study on the subject to date, reliable information on disease progression, treatment options, and the risks involved should be available to these women. It is too early to define fertility preservation as the standard of care for all women with endometriosis, as few cases have been reported and the available data do not allow for adequate robust cost-utility analyzes. However, fertility preservation should be considered for those with unoperated bilateral endometriomas and for those who have previously removed unilateral endometriomas and need surgery for a contralateral recurrence. Furthermore, age is currently the most important prognostic factor associated with fertility. Available strategies include cryopreservation of embryos and oocytes, and women should be counseled individually about the risks, benefits and costs involved. In this scenario, the management of endometriosis by a multidisciplinary team is a fundamental step towards the achievement of successful results.

National Specialty Commission in Endometriosis of the Brazilian Federation of Gynecology and Obstetrics Associations (FEBRASGO)

President:

Julio Cesar Rosa e Silva

Vice-President:

Helizabet Salomão Abdalla

Secretary:

Márcia Mendonça Carneiro

Members:

Carlos Alberto Petta

Carlos Augusto Pires Costa Lino

Corival Lisboa Alves de Castro

Eduardo Schor

Joao Nogueira Neto

João Sabino Lahorgue da Cunha Filho

Marco Aurélio Pinho de Oliveira

Marcos Tcherniakovsky

Maurício Simões Abrão

Omero Benedicto Poli Neto

Ricardo de Almeida Quintairos

Sidney Pearce
